# The role of matrix metalloproteinases in the modulation of aqueous humor in glaucoma patients

**DOI:** 10.22336/rjo.2025.33

**Published:** 2025

**Authors:** Cerbulescu Teodor, Barac Ileana Ramona, Boruga Ovidiu, Sălăvăţ Mădălina Casiana, Leuştean Laurenţiu, Duncă Ştefan Daciana, Calancea Andrei, Barac Andreea Diana

**Affiliations:** 1 “Victor Babeş” University of Medicine and Pharmacy, Timişoara, Romania; 2 “Carol Davila” University of Medicine and Pharmacy, Bucharest, Romania; 3 “Iuliu Hațieganu” University of Medicine and Pharmacy, Cluj, Romania

**Keywords:** matrix metalloproteinases, glaucoma, aqueous humor dynamics, intraocular pressure, extracellular matrix remodeling, ECM = extracellular matrix, MMP = Matrix Metalloproteinase, TIMP = Tissue Inhibitor of Metalloproteinase, IOP = Intraocular Pressure, JEP = Journal of Experimental Pharmacology, OPTH = Clinical Ophthalmology, PMID = PubMed Identifier, PMCID = PubMed Central Identifier

## Abstract

Matrix metalloproteinases are crucial proteolytic enzymes involved in the degradation and remodeling of the extracellular matrix within the ocular structures. Their expression and activity significantly influence aqueous humor dynamics and intraocular pressure in patients with glaucoma. Elevated intraocular pressure, a significant risk factor for glaucoma, is often associated with disturbed aqueous humor outflow.

This review summarizes current findings on the role of matrix metalloproteinases in modulating aqueous humor and their potential as therapeutic targets in the treatment of glaucoma.

## Introduction

Glaucoma is a leading cause of blindness globally, affecting millions of individuals. The pathophysiology of glaucoma primarily involves neurodegeneration of retinal ganglion cells and alterations in aqueous humor outflow. MMPs are a family of enzymes that degrade extracellular matrix (ECM) components, and their activity is balanced by tissue inhibitors of metalloproteinases (TIMPs). Changes in the MMP/TIMP ratio can contribute to elevated intraocular pressure (IOP), thereby worsening the glaucomatous process. Understanding the mechanisms by which matrix metalloproteinases (MMPs) affect aqueous humor dynamics may offer insights into innovative therapeutic approaches [1-3].

## Materials and methods

A comprehensive literature review was conducted to examine the role of MMPs in the modulation of aqueous humor in glaucoma patients. The methodology involved the following steps:

1. Data Sources: A systematic search was performed across several scientific databases, including PubMed, Google Scholar, and Scopus. The search terms included “matrix metalloproteinases”, “aqueous humor”, “glaucoma”, “IOP”, and “trabecular meshwork”.
2. Selection Criteria: Inclusion criteria were established to focus on studies published in peer-reviewed journals over the last decade (2013-2023). Only studies involving human subjects or relevant animal models were considered for review. Both clinical trials and observational studies that explored the relationship between matrix metalloproteinase (MMP) activity and aqueous humor dynamics were included in the analysis.
3. Data Extraction: Relevant data regarding MMP types, their expression levels, and the associated effects on intraocular pressure (IOP) and aqueous humor outflow were extracted. This included basic demographics of the study populations, methodologies used for measuring IOP, and quantitative values of MMP expression (e.g., enzyme activity assays, immunohistochemistry, and tissue-level evaluations).
4. Analysis of Findings: Studies were analyzed for their findings on MMP and TIMP levels in the context of glaucoma. The results of various therapeutic interventions, particularly those involving ocular hypotensive agents such as prostaglandin analogs, and their effects on matrix metalloproteinase (MMP) expression in the trabecular meshwork were meticulously reviewed.
5. Synthesis of Results: The findings from the selected studies were synthesized into a cohesive narrative to establish the current understanding of how matrix metalloproteinases (MMPs) influence the modulation of aqueous humor. This synthesis allowed for the identification of common insights, discrepancies, and research gaps in the field.

## Results

The analysis of the literature revealed several key findings regarding the role of MMPs in aqueous humor dynamics and their correlation with glaucoma:

1. Increased MMP Expression in Glaucoma: Numerous studies have reported that glaucoma patients exhibit elevated levels of specific matrix metalloproteinases (MMPs), particularly MMP-2 and MMP-9, within the aqueous humor and trabecular meshwork. This increase is associated with altered aqueous outflow and elevated intraocular pressure (IOP) [1,3,4].

2. Correlation with Intraocular Pressure: A clear correlation was observed between MMP activity and intraocular pressure (IOP). Elevated MMP levels have been linked to increased IOP in both human studies and animal models. Specifically, patients with higher expressions of MMP-9 showed significantly higher IOP readings compared to those with lower levels [1,5].

3. Effects of Ocular Hypotensive Agents: Treatment with ocular hypotensive medications, particularly prostaglandin analogs, resulted in a notable increase in MMP activity. For example, one study found that administering latanoprost resulted in the upregulation of MMP-2, thereby enhancing aqueous humor outflow and effectively lowering intraocular pressure (IOP) [2,4-6].

4. Role in Extracellular Matrix Remodeling: MMPs were shown to play a critical role in the remodeling of the extracellular matrix within the trabecular meshwork. Studies utilizing immunohistochemical techniques demonstrated that MMPs facilitate ECM degradation, thus reducing resistance to aqueous humor outflow. Consequently, this remodeling process is believed to play a role in maintaining normal intraocular pressure (IOP) levels [3].

5. Imbalance with TIMPs: A consistent finding across various studies was the altered balance between matrix metalloproteinases (MMPs) and tissue inhibitors of metalloproteinases (TIMPs) in patients with glaucoma. Increased levels of MMPs were often accompanied by decreased TIMP expression, suggesting that this imbalance contributes to increased outflow resistance and higher intraocular pressure (IOP) [5].

6. Emerging Therapeutic Targets: Finally, the literature highlights the potential for MMPs as therapeutic targets. Studies have proposed that modulating matrix metalloproteinase (MMP) activity could be a novel strategy for managing glaucoma, specifically by enhancing aqueous outflow and lowering intraocular pressure (IOP) through the timed administration of specific MMP inhibitors or agents that upregulate beneficial MMPs [5].

7. Effects of Anti-Glaucomatous Medications on Matrix Metalloproteinase (MMP) Levels: Anti-glaucomatous medications have been shown to significantly influence the expression and activity of matrix metalloproteinase (MMP). Studies highlight the following effects: Prostaglandin Analogs: Treatment with prostaglandin analogs, such as latanoprost and travoprost, was found to upregulate MMP-2 and MMP-9 levels in the aqueous humor. For instance, a study by Jayanetti et al. (2020) reported that latanoprost increased MMP-2 expression by approximately 50% in the trabecular meshwork, enhancing aqueous humor outflow and effectively reducing IOP by an average of 15-20% [5].

a) Beta-Blockers: Timolol, a common beta-blocker used in glaucoma management, exhibited a mixed influence on MMP expression. Research indicates that timolol administration led to a modest reduction in MMP-9 levels, potentially contributing to its lower efficacy in enhancing aqueous outflow compared to prostaglandin analogs. Specifically, one study observed a 25% decrease in MMP-9 levels after six weeks of timolol therapy in patients with glaucoma [6].

b) Alpha Agonists: Brimonidine, an alpha-agonist, has been associated with an increase in TIMP-1 levels while also affecting MMP expression. A study revealed that brimonidine treatment resulted in a 30% increase in MMP-2 levels alongside a corresponding increase in TIMP-1, indicating a potential interaction that stabilizes ECM remodeling while aiding in IOP control [5].

c) Carbonic Anhydrase Inhibitors: Treatments such as dorzolamide, while primarily functioning to reduce aqueous humor production, have also shown a correlation with decreased MMP activity in some cases. A study reported a 15% reduction in MMP-9 activity following five weeks of dorzolamide treatment, suggesting a role in preserving extracellular matrix (ECM) integrity and potentially mitigating glaucomatous progression [5].

d) These findings illustrate that while some anti-glaucomatous medications enhance matrix metalloproteinase (MMP) activity to promote aqueous humor outflow, others can either stabilize or inhibit MMP activity, underscoring the complexity of pharmacological interactions within glaucoma treatment.

## Discussion

The findings from the literature suggest that matrix metalloproteinases (MMPs) play a crucial role in modulating aqueous humor dynamics and regulating intraocular pressure (IOP) in patients with glaucoma. This review highlights several critical implications for understanding the pathology of glaucoma and developing therapeutic strategies. Pathophysiological Implications:

1. The observed increase in MMP activity in glaucoma patients highlights the crucial role these enzymes play in remodeling the extracellular matrix (ECM) within the trabecular meshwork. MMPs facilitate the degradation of ECM components, which helps reduce resistance to aqueous humor outflow, a crucial step in maintaining normal intraocular pressure (IOP) levels. However, the intricate balance between MMPs and TIMPs is essential; an imbalance, as seen in glaucoma, can lead to increased outflow resistance and higher IOP, exacerbating the disease process [6].

2. Clinical Relevance of MMPs as Biomarkers: Elevated levels of specific matrix metalloproteinases (MMPs), particularly MMP-2 and MMP-9, may serve as potential biomarkers for the progression of glaucoma. Monitoring MMP levels in the aqueous humor might provide valuable insights into the severity of the disease and the responsiveness to treatment, offering a personalized approach to glaucoma management [7].

3. Influence of Anti-Glaucomatous Medications: The review highlights the varied effects of anti-glaucomatous medications on matrix metalloproteinase (MMP) activity, underscoring their complex role in therapy. Prostaglandin analogs are particularly noteworthy for their ability to enhance matrix metalloproteinase (MMP) activity, thereby promoting aqueous outflow and achieving IOP reduction. Conversely, beta-blockers like timolol show a more nuanced impact, potentially leading to reduced MMP expression and lower efficacy in this regard. Understanding these dynamics enables clinicians to tailor treatment regimens based on the specific modulation effects of MMPs, potentially improving patient outcomes.

4. Therapeutic Targets: The evidence supporting the role of MMPs as therapeutic targets is promising. Modulating MMP activity presents a novel pharmacological strategy for enhancing aqueous humor outflow and reducing intraocular pressure (IOP). Current research suggests that targeted MMP inhibitors or agents that can upregulate beneficial MMPs may be effective in managing glaucoma, particularly in patients who are unresponsive to traditional treatments. Optimizing treatment protocols involving MMP modulation will require extensive clinical trials to evaluate safety and efficacy.

5. Future Directions: Future research should focus on elucidating the precise mechanisms by which matrix metalloproteinases (MMPs) influence extracellular matrix (ECM) remodeling and the dynamics of aqueous humor. Understanding the signaling pathways involved in matrix metalloproteinase (MMP) expression and activity can lead to the development of new therapeutic agents. Furthermore, investigating the relationship between lifestyle factors, such as smoking cessation, and their effects on MMP levels may yield additional insights into glaucoma management [8].

6. Limitations and Gaps: While the current findings provide a foundation for understanding the role of MMPs in glaucoma, there are still knowledge gaps that need to be addressed. More standardized methods for measuring MMP activity and expression levels are required to validate findings across studies. Additionally, long-term studies investigating the effects of MMP-targeted therapies in diverse patient populations will be necessary to fully realize their therapeutic potential.

## Conclusion

In conclusion, matrix metalloproteinases play a crucial role in the pathophysiology of glaucoma, influencing the dynamics of aqueous humor and regulating intraocular pressure (IOP). Anti-glaucomatous medications modulate matrix metalloproteinase (MMP) activity, highlighting their potential as therapeutic targets. Future strategies aimed at optimizing MMP modulation may pave the way for more effective glaucoma management, ultimately reducing the burden of this debilitating eye disease.
